# How Quickly Can a Rat Perceive Novel Odors?

**DOI:** 10.1371/journal.pbio.0060094

**Published:** 2008-04-08

**Authors:** Rachel Jones

Odor perception in mammals is a multistep process that begins when olfactory sensory neurons in the animal's nose detect an odor molecule and then transmit that sensory information as an electrical signal to the brain. The first brain area to receive these signals is the olfactory bulb, where the sensory neurons end in small structures called glomeruli. Olfactory cues trigger complex patterns of activity both in the olfactory sensory neurons and in the glomeruli within the brain. How these complex patterns underlie an animal's ability to sense and respond to odors remains obscure. In a new study, Daniel Wesson et al. shed light on how these early signals lead to odor perception and subsequent behavioral changes by investigating how quickly a rat can respond to a novel odor. The speed of response, they show, depends on how quickly the olfactory bulb receives neural information about such an odor from the sensory neurons.

The authors combined two techniques in their study. To investigate the speed of the rats' behavioral responses to odors, the authors monitored a natural, spontaneous response known as exploratory sniffing. When a rat encounters a new odor for the first time, it responds by sniffing more rapidly, and the authors measured how soon this behavior began after a new odor was first inhaled. The second technique involved optical imaging and used calcium-sensitive dyes to visualize neural activity. Using this method, the authors could determine when action potentials from the olfactory sensory neurons first arrived at the olfactory bulb. By combining both of these techniques, Wesson et al. were able to gain insight into both the speed with which the initial neural processing of an odor takes place and how quickly this can be translated into a behavioral response by central and motor processes.

The time between the initial inhalation of a novel odor and the onset of exploratory sniffing was surprisingly short—as little as 140 ms and usually less than 200 ms. The optical imaging showed that neural input from the receptor neurons arrives at the olfactory bulb glomeruli between 100 and 150 ms after the beginning of the initial inhalation, leaving very little time—between 50 and 100 ms—for central and motor processing to lead to the behavioral response. This figure, allowing time for the transmission of an action potential along the axon to the olfactory bulb, also indicates that an olfactory sensory neuron can generate an action potential in response to an odor as little as 70 ms after inhalation begins—much faster than has been found in isolated olfactory neurons.

**Figure:**
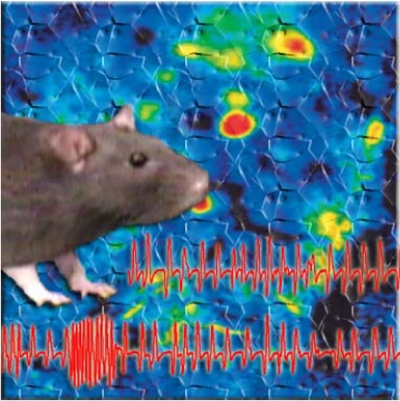
Increasing evidence highlights the importance of sniffing behavior in olfactory coding after the olfactory receptor sheet within the nose is exposed to an odor molecule (odorant). The respiratory trace (red, above) of a rat recorded during odor discriminations is overlaid on a main olfactory bulb calcium response map (below) of the odorant eugenol (from clove oil).

When receptor inputs arrive at the olfactory bulb after exposure to a particular odor, they lead to a distinct spatial pattern of activation across the different glomeruli in the bulb, which also develops over time in an odor-specific manner. It has been hard to identify precisely how these spatial and temporal patterns of activation are related to odor perception and the initiation of a behavioral response. This study shows that the behavioral response occurs too quickly for it to depend on the full temporal development of activation in the olfactory bulb. In most trials, exploratory sniffing began before the glomeruli in the bulb had reached their peak of activation, showing that only the initial activation pattern is needed for a rat to identify an odor as novel.

Based on these results, the authors conclude that odorant identity is likely to be encoded by the sequence of activation of glomeruli (rather than the pattern of peak activation or later temporal features). Alternatively, the earliest-activated glomeruli—which are not necessarily the most strongly activated overall—might contribute preferentially to the coding of odorant identity. Later developments in the pattern of olfactory bulb activation are more likely to contribute to more demanding olfactory tasks than simply identifying a single odor as novel or not, such as integrating multiple odors in a natural environment. Clearly, the combination of behavioral techniques with optical imaging provides powerful data with which to constrain models of sensory processing.

